# Super-Resolution Community Detection for Layer-Aggregated Multilayer Networks

**DOI:** 10.1103/PhysRevX.7.031056

**Published:** 2017-09-26

**Authors:** Dane Taylor, Rajmonda S. Caceres, Peter J. Mucha

**Affiliations:** 1 Carolina Center for Interdisciplinary Applied Mathematics, Department of Mathematics, University of North Carolina, Chapel Hill, North Carolina 27599, USA; 2 Department of Mathematics, University at Buffalo, State University of New York, Buffalo, New York 14260, USA; 3 Lincoln Laboratory, Massachusetts Institute of Technology, Lexington, Massachusetts 02420, USA

**Keywords:** Complex Systems, Interdisciplinary Physics, Statistical Physics

## Abstract

Applied network science often involves preprocessing network data before applying a network-analysis method, and there is typically a theoretical disconnect between these steps. For example, it is common to aggregate time-varying network data into windows prior to analysis, and the trade-offs of this preprocessing are not well understood. Focusing on the problem of detecting small communities in multilayer networks, we study the effects of layer aggregation by developing random-matrix theory for modularity matrices associated with layer-aggregated networks with *N* nodes and *L* layers, which are drawn from an ensemble of Erdős–Rényi networks with communities planted in subsets of layers. We study phase transitions in which eigenvectors localize onto communities (allowing their detection) and which occur for a given community provided its size surpasses a detectability limit K^*^. When layers are aggregated via a summation, we obtain $K^{*}\propto O(\sqrt{NL}/T)$, where *T* is the number of layers across which the community persists. Interestingly, if *T* is allowed to vary with *L*, then summation-based layer aggregation enhances small-community detection even if the community persists across a vanishing fraction of layers, provided that *T/L* decays more slowly than 𝒪(*L*^−1/2^). Moreover, we find that thresholding the summation can, in some cases, cause K^*^ to decay exponentially, decreasing by orders of magnitude in a phenomenon we call super-resolution community detection. In other words, layer aggregation with thresholding is a nonlinear data filter enabling detection of communities that are otherwise too small to detect. Importantly, different thresholds generally enhance the detectability of communities having different properties, illustrating that community detection can be obscured if one analyzes network data using a single threshold.

## I. INTRODUCTION

**FIG. 1:**
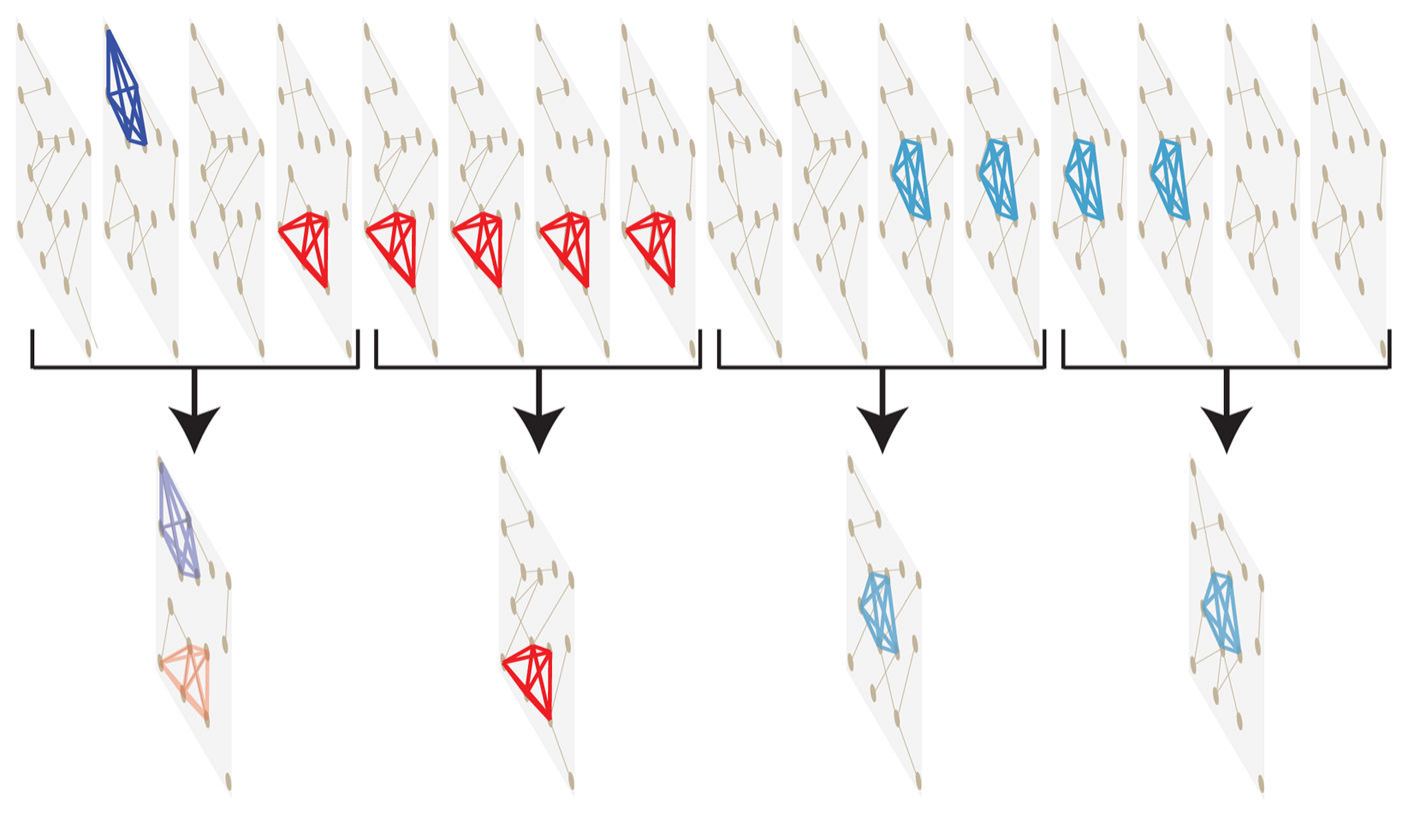
Preprocessing networks (including multilayer representations of temporal networks) often involves aggregating network data into bins (or time windows). We study how many layers must contain a community in order for aggregation to enhance its detection and introduce layer aggregation with thresholding as a filter enabling super-resolution community detection.

Network-based modeling provides a powerful framework for analyzing high-dimensional data sets and complex systems [1]. Often, a network is best represented by a set of network layers that encode different types of interactions, such as categorical social ties [2] or a network at different instances in time [3], and an important pursuit involves extending network theory to the multilayer setting [4,5]. Sometimes, however, a multilayer framework can require too much computational overhead or can represent an over-modeling (e.g., when the layers are correlated, either in terms of the edge overlap [6] or other properties [7–9]), and it can be beneficial to aggregate layers [9–11]. In particular, aggregation provides a crucial step for analyzing temporal network data, which is often binned into time windows [12,13] (see Fig. 1). Layer aggregation and other types of network preprocessing (e.g., sparsification [14], network inference [15], and denoising [16,17]) can greatly influence the resulting network structure, which in turn influences the outcomes of network analyses and their many applications. In general, there remains a significant need for improved theoretical understanding for how such network preprocessing influences network-analysis methodology.

We study the effects of layer aggregation on community detection, one of the widely used methods for studying social, biological, and physical networks [18–21]. Communities are typically studied as dense subgraphs and can represent, for example, coordinating neurons in the brain [13] or a social clique [22] in a social network. (Hereafter, we restrict our usage of the term “clique” to the graph-theoretical meaning of a subgraph with all-to-all coupling.) Of particular interest is the detection of small-scale communities, which is a paradigmatic pursuit for anomaly detection within the fields of signal processing and cybersecurity [23–28]. In this context, small communities can represent anomalous events such as attacks [23], intrusions [24], and fraud [25].

Given these and many other applications, there is great interest in understanding fundamental limitations on community detection [11,26–36]. We highlight recent detectability results for multilayer [10,11,37] and temporal networks [29]. It is worth noting that much of the detectability research has focused on large-scale communities whose sizes are 𝒪(*N*), where *N* is the number of nodes in the network [29–35], and the phase transitions are typically driven by varying the prevalence (e.g., edge density) of the communities. In contrast, detectability phase transitions for small communities can also be onset by varying their size *K* [11,26–28] and are thus a type of resolution limit [36]. We note that the literatures on detectability and resolution limits have developed independently, and there is need for a better understanding of the relationship between these topics. In particular, a planted clique in a single-layer Erdős-Rényi (ER) network is detectable via a spectral analysis only if its size *K* surpasses a detectability limit $K^{*}\propto O(\sqrt{N})$ [26], in which case, a dominant eigenvector (in this case, that corresponding to the second-largest eigenvalue of the adjacency matrix) localizes onto the clique. Extending previous research for the detectability of a clique planted in single-layer networks [26–28] and a clique that persists across all layers of a multilayer network [11], herein we study the detectability of small communities (including, but not limit to, cliques) planted in a subset of layers in a multilayer network.

With the application of detecting small communities in mind, we study the effects of layer aggregation as a network preprocessing step. We first ask a foundational question: Across how many layers must a community persist in order for layer aggregation to benefit detection. To this end, we study a multilayer network model in which small communities are hidden in network layers generated as ER networks with *N* nodes and *L* layers with (possibly) heterogeneous edge probabilities. We study detectability phase transitions wherein eigenvectors localize onto communities, which we analyze by developing random matrix theory for the eigenvectors of modularity matrices associated with an aggregation of the layers. When the aggregation is given by summation of the adjacency matrices, the detectability phase transition occurs when a community’s size *K* ≪ *N* surpasses a critical value $K^{*}\propto \sqrt{NL}/T$, where *T* is the number of layers across which a community persists. Note that if *T* depends on *L*, then summation-based layer aggregation benefits small-community detection even if the fraction *T/L* of layers containing the community vanishes, provided that the fraction decays more slowly than 𝒪(*L*^−1/2^).

We additionally study network preprocessing via thresholding—that is, we threshold a summation of layers’ adjacency matrices at some value *L̃* so that there exists an unweighted edge between two nodes in the aggregated network if and only if there exists at least *L̃* edges between them across the *L* layers. While it is well known that thresholding can be used to simultaneously sparsify and dichotomize a network, here we introduce thresholding as a nonlinear data filter [38] for enhancing small-community detection. Specifically, we find that thresholding can, in some cases, reduce K^*^ by orders of magnitude, revealing communities that are otherwise too small to detect. We call this phenomenon super-resolution community detection and show, for clique detection in sparse networks, that K^*^ decays exponentially with $\sqrt{L}/T$ for threshold *L̃* = *T*. Importantly, we find that different thresholds enhance the detection of communities with different properties (e.g., size and edge density), illustrating how community structure can be obscured if one uses a single threshold, which is an important insight for network preprocessing in general.

The remainder of this paper is organized as follows. In Sec. II, we further specify our model. In Sec. III, we study the effects of layer aggregation on detectability phase transitions characterized by eigenvector localization. In Sec. IV, we highlight implications of our findings with a numerical experiment involving small-community detection in a temporal network. We provide a discussion in Sec. V

## II. MODEL

### A. Multilayer networks with planted small communities

We generate *L* network layers with *N* nodes so that each layer *l* ∈ {1,…, *L*} is an ER random graph with edge probability *p_l_* ∈ (0, 1), which is allowed to vary across the layers. We plant *R* communities via the following process. For *r* ∈ {1…, *R*}, uniformly at random, we select a set 𝒯*_r_* ⊂ {1,…, *L*} of layers and a set 𝒦*_r_* ⊂ 𝒱 = {1,…, *N*} of nodes, and we define an edge probability *ρ_r_*. The variable *K_r_* = |𝒦*_r_*| ≪ *N* denotes the size of community *r*, and we refer to *T_r_* = |𝒯 *_r_*| as its persistence across network layers. Then, for each *r*, we construct a dense subgraph between nodes 𝒦*_r_* in layers 𝒯*_r_* by first removing edges between them occurring under the ER model and creating new edges with probability *ρ_r_*. To ensure that the communities are denser than the remaining network, we assume *ρ_r_ >* 〈*p_l_*〉, where 〈·〉 denotes the mean value across all layers. We allow self-edges in both the ER model and the planted communities. We note that the layers are not required to have a particular ordering, and the community is not restricted only to consecutive layers. Moreover, we restrict our study to nonoverlapping communities by assuming that the communities involve different nodes so that 𝒦*_r_* ∩ 𝒦*_s_* = 0 for any *r* ≠ *s*. We leave open the study of eigenvector localization in the case of overlapping communities. Finally, we assume Σ*_r_K_r_* ≪ *N* so that only a small fraction of nodes are involved in communities, making them anomalous structures.

### B. Layer-aggregation methods

We find that layer aggregation is a preprocessing step for multilayer networks that can be used to reduce data size and/or as a data filter to benefit network-analysis outcomes such as community detection. Following the approach in Ref. [10], we study two methods for aggregating layers of a multilayer network:

- The summation network corresponds to the weighted adjacency matrix **Ā** = Σ*_l_***A**^(^*^l^*^)^, where **A**^(^*^l^*^)^ denotes the symmetric adjacency matrix encoding each network layer *l* ∈ {1,…, *L*}.
- The family of thresholded networks represented by unweighted adjacency matrices {**Â**^(^*^L̃^*^)^} are obtained by applying a threshold *L̃* ∈ {1,…, *L*} to the entries {*Ā_ij_*} of matrix **Ā**,
  (1) $${\hat{A}}_{ij}^{\left(\tilde{L}\right)}=\left\{ \begin{array}{ll} 1 & \text{if}\;{\bar{A}}_{ij}\geq \tilde{L}\\ 0 & \text{otherwise.} \end{array}\right.$$

Note that thresholding dichotomizes the network, and one can vary *L̃* to tunably sparsify the network.

## III. DETECTABILITY OF SMALL COMMUNITIES WITH EIGENVECTOR LOCALIZATION

We now develop random matrix theory to analyze how layer aggregation affects small-community detection. In Sec. III A, we present results for aggregation by summation, studying the fraction of layers that must contain a community in order for layer aggregation to enhance detection. In Sec. III B, we present results for layer aggregation with thresholding, highlighting that certain threshold values can yield super-resolution community detection.

### A. Layer aggregation via summation

#### 1. Random matrix theory for modularity matrices

We first describe the statistical properties of matrix entries {*Ā_ij_*}. For edges (*i, j*)∈∉*_r_*{𝒦*_r_* × 𝒦*_r_*}, {*Ā_ij_*} are independent and identically distributed (i.i.d.) random variables following a Poisson binomial distribution, *P*(*Ā_ij_* = *a*) = *f*_PB_(*a; L,* {*p_l_*}), where

(2) $$f_{\text{PB}}(a;L,\{p_{l}\})=\sum_{S\in S_{a}}\prod_{l\in S}p_{l}\prod_{m\in \{1,\ldots ,L\}\backslash S}\left(1-p_{m}\right),$$

and 𝒮*_a_* denotes the set of $\left(\begin{array}{c} L\\ a \end{array}\right)$different subsets of layers {1,…, *L*} that have cardinality *a* (i.e., 𝒮_1_ ={{1},{2},…}, 𝒮_2_ = {{1, 2}, {1, 3},…}, and so on). We note that *f*_PB_(*a; L,* {*p_l_*}) has mean *L*〈*p_l_*〉 and variance *L*〈*p_l_*(1 − *p_l_*)〉. When the edge probability is identical across the layers (i.e., *p_l_* = *p*), then Eq. (2) simplifies to the binomial distribution,

(3) $$f(a;L,p)=\left(\begin{array}{c} L\\ a \end{array}\right)\;p^{a}{\left(1-p\right)}^{P-a},$$

with mean *Lp* and variance *Lp*(1 − *p*).

For within-community edges (*i, j*) ∈ {𝒦*_r_* × 𝒦*_r_*} associated with community *r*, the entries {*Ā_ij_*} are i.i.d. random variables following $f_{\text{PB}}(a;L,\{q_{l}^{\left(r\right)}\})$, where $q_{l}^{\left(r\right)}=\rho_{r}$ for *l* ∈ 𝒯*_r_* and otherwise $q_{l}^{\left(r\right)}=p_{l}$. It follows that the entries have mean *T_r_ρ_r_* +Σ_*l*∈{1,…, *L*}\𝒯 _*r*__*p_l_* and variance *T_r_ρ_r_*(1−*ρ_r_*)+Σ_*l*∈{1,…, *L*}\𝒯 _*r*__*p_l_*(1−*p_l_*). Because the layers 𝒯*_r_* are selected uniformly at random, the expected mean and variance across all possible choices for 𝒯*_r_* are given by *T_r_ρ_r_* + (*L* − *T_r_*)〈*p_l_*〉 and *T_r_ρ_r_*(1 − *ρ_r_*) + (*L* − *T_r_*)〈*p_l_*(1 − *p_l_*)〉, respectively.

We now study the spectra of the modularity matrix [39],

(4) $$\bar{B}=\bar{A}-L\langle p_{i}\rangle 11^{T},$$

based on an ER null model in which each edge has expected weight *L*〈*p_i_*〉. Importantly, this null model does not use knowledge that edges (*i, j*) between nodes *i*, *j* ∈ 𝒦*_r_* have different expected edge probability [i.e., *T_r_ρ* + (*L* − *T_r_*)〈*p_i_*〉 vs *L*〈*p_i_*〉], which respects our assumption that it is unknown which nodes are in the hidden community. We note that one could also define the ER null model with the observed mean edge probability $L\langle p_{i}\rangle +\sum_{r}\left[(K_{r}^{2}T_{r})/N^{2}L](\rho_{r}-\langle p_{i}\rangle \right)$ to account for the slight increase in overall edge probability due to the presence of small communities. However, this change does not affect the position of the dominant eigenvalues relative to the bulk, which is the relevant issue for community detectability, as we will see below. In particular, since $|(K_{r}^{2}T_{r})/N^{2}L|\;\ll 1$ for each *r*, even the shift of the single associated eigenvalue within the bulk is negligible; therefore, we focus on the null model with expected edge weight *L*〈*p_i_*〉.

We develop random matrix theory based on the analysis in Refs. [27,40]. To this end, we note that **B̄** can be written in the form

(5) $$\bar{B}=\langle \bar{B}\rangle +X,$$

where

(6) $$\langle \bar{B}\rangle =\sum_{r}\theta_{r}u^{\left(r\right)}{\left(u^{\left(r\right)}\right)}^{T}$$

is a rank-*R* matrix with eigenvalues given by

(7) $$\theta_{r}=T_{r}K_{r}(\rho_{r}-\langle p_{l}\rangle ),$$

and {**u**^(^*^r^*^)^} are normalized indicator vectors for the *R* communities that have entries

(8) $$u_{i}^{\left(r\right)}=\left\{ \begin{array}{ll} \sqrt{1/K_{r}} & i\in K_{r}\\ 0 & \text{otherwise.} \end{array}\right.$$

The random matrix **X** has zero-mean entries *X_ij_* with variance *Tρ_r_*(1−*ρ_r_*)+(*L*–*T_r_*)〈*p_l_*(1–*p_l_*)〉 if (*i,j*)∈𝒦*_r_*×𝒦*_r_*, and *L*〈*p_l_*(1 − *p_l_*)〉 otherwise. In the *N* → ∞ limit, and assuming the sizes {*K_r_*} grow more slowly than *N*, then the $\sum_{r}K_{r}^{2}\ll N^{2}$ matrix entries corresponding to communities become negligible and **X** limits to a Wigner matrix [41]. This allows us to use known results for the limiting dominant eigenvector of low-rank perturbations of Wigner matrices with variance 1/*N*. Specifically, we define $\gamma =1/\sqrt{NL\langle p_{l}(1-p_{l})\rangle }$ so that the matrix *γ***X** has entries with variance 1/*N* in the limit. We similarly define

(9) $${\bar{\theta }}_{r}=\gamma \theta_{r}=\frac{T_{r}K_{r}}{\sqrt{NL}}\frac{\rho_{r}-\langle p_{l}\rangle }{\sqrt{\langle p_{l}(1-p_{l})\rangle }}$$

so that *γ***B̄** = Σ*_r_θ̄_r_***u**^(^*^r^*^)^(**u**^(^*^r^*^)^)*^T^* + *γ***X**. It follows that the limiting *N* → ∞ dominant eigenvectors {**v**^(^*^r^*^)^} of *γ***B̄** (and of **B̄** since scalar multiplication does not affect eigenvectors) satisfy [40,42]

(10) $${|\langle v^{\left(r\right)},u^{\left(r\right)}\rangle |}^{2}=\left\{ \begin{array}{ll} 1-1/{\bar{\theta }}^{2} & \bar{\theta }>1\\ 0 & \text{otherwise.} \end{array}\right.$$

Note we assume that the dominant eigenvectors have been suitably enumerated so that **v**^(^*^r^*^)^ corresponds to the eigenvector localizing on community *r*. The value *θ̄_r_* = 1 identifies critical points at which there is a phase transition in eigenvector localization and detectability for community *r*, and this gives the critical community size

(11) $$K_{r}^{*}=\sqrt{T_{r}^{-2}NL}\frac{\sqrt{\langle p_{l}(1-p_{l})\rangle }}{\rho_{r}-\langle p_{l}\rangle }.$$

In other words, a small community can be detected using a dominant vector **v**^(^*^r^*^)^ of **B̄** only when $K_{r}>K_{r}^{*}$. We note that setting *L* = *T_r_* = 1, *ρ_r_* = 1, and *p_l_* = *p* in Eq. (11) recovers $K_{r}^{*}=\sqrt{Np/(1-p)}$, which describes the detectability transition for a single planted clique in a single-layer network [26].

We highlight an important consequence of Eq. (11). First, if the community persists across some fixed fraction of the layers, *T*(*L*) = *cL*, then $K_{r}^{*}\propto \sqrt{N/L}$; therefore, if *N*, *p*, and *T_r_/L* are held fixed and *L* increases, then $K_{r}^{*}$ vanishes with scaling 𝒪(*L*^−1/2^). This square-root scaling behavior is similar to that obtained for detection in layer aggregation of large-scale communities that persist across all layers [10]. Second, for fixed *N* and *p*, a community of fixed size *K_r_* and persistence *T_r_* will become impossible to detect as *L* increases because $K_{r}^{*}$ increases with scaling 𝒪(*L*^1/2^). This result highlights the importance of knowing which layers potentially contain the community since the aggregation of layers lacking the community can severely inhibit its detection.

Digging further, one can let *T_r_* vary with *L* and then ask how $K_{r}^{*}$ depends on the scaling behavior for *T_r_*. For *T_r_* ∝ *L^β^*, Eq. (11) implies $K_{r}^{*}\propto L^{1/2-\beta }$ so that as *L* → ∞,

(12) $$K_{r}^{*}\to \left\{ \begin{array}{ll} 0 & \beta >1/2\\ \infty & \beta <1/2. \end{array}\right.$$

In other words, *T_r_*, the number of layers containing the community, must increase with *L* at least as 𝒪(*L*^1/2^); otherwise, summation-based layer aggregating will inhibit (rather than promote) small-community detection. Note that *T* ∝ *L*^−1/2^ is a critical case in which $K_{r}^{*}$ is independent of *L*. We highlight that Eq. (12) is somewhat surprising since summation-based aggregation benefits detection even if the fraction *T_r_/L* of layers containing the community vanishes with *L*, provided that it decays more slowly than 𝒪(*L*^−1/2^).

#### 2. Numerical validation and scaling behavior

**FIG. 2:**
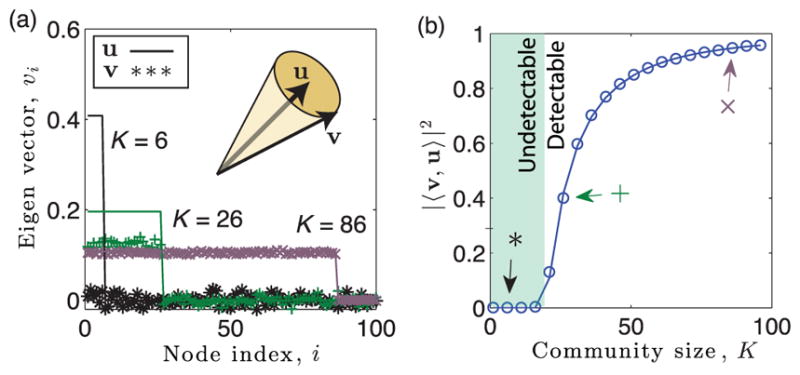
Eigenvector localization yields detectability phase transition. (a) Entries $v_{i}^{\left(r\right)}$ (symbols) of a dominant eigenvector of the modularity matrix for the summation network of a multilayer network with a hidden community of size *K_r_*. Parameters include *T_r_* = 2, *L* = 16, *N* = 10^4^, *ρ* = 1, and the edge probabilities {*p_l_*} of layers are Gaussian distributed with mean 〈*p_l_*〉 = 0.01 and standard deviation *σ_p_* = 0.001. To allow visualization, we assume nodes *i* ∈ {1,…, *K*} are in the community, and we only visualize $v_{i}^{\left(r\right)}$ for nodes *i* ∈ {1, 100}. As shown by the illustration, as *K_r_* increases, **v**^(^*^r^*^)^ aligns with the indicator vector **u**^(^*^r^*^)^, which is nonzero only for the *K_r_* ≪ *N* entries $u_{i}^{\left(r\right)}$ that correspond to nodes in the community, 𝒦*_r_*. (b) Observed (symbols) and predicted (curves) values of |〈**v**^(^*^r^*^)^, **u**^(^*^r^*^)^〉|^2^ given by Eq. (10) quantify this localization phenomenon. Arrows indicate the values of *K* used for panel (a). The critical size $K_{r}^{*}$ such that |〈**v**^(^*^r^*^)^, **u**^(^*^r^*^)^〉|^2^ = 0 for $K_{r}\leq K_{r}^{*}$, whereas |〈**v**^(^*^r^*^)^, **u**^(^*^r^*^)^〉|^2^ *>* 0 for $K_{r}>K_{r}^{*}$ marks a phase transition—that is, both in terms of eigenvector localization and detectability of the community.

We support Eqs. (10) and (11) in Fig. 2, using numerical experiments with *N* = 10^4^ nodes and edge probabilities {*p_l_*} drawn from a Gaussian distribution with mean *p* = 0.01 and standard deviation *σ_p_* = 0.001. We focus on the case of clique detection (i.e., *ρ* = 1), hiding the clique in *T* = 2 of the *L* = 16 layers. In Fig. 2(a), we plot the entries { $v_{i}^{\left(r\right)}$} (symbols) of the dominant eigenvector of the modularity matrix for the summation network as well as the entries { $u_{i}^{\left(r\right)}$} for the indicator vector, which are nonzero only for nodes *i* ∈ 𝒦 involved in the clique. We show results for community sizes *K_r_* ∈ {6, 26, 86}, which respectively place the system below, just above, and well above the phase transition. The illustration highlights that as *K* increases, vector **v**^(^*^r^*^)^ aligns with **u**^(^*^r^*^)^.We quantify this localization phenomenon by plotting in Fig. 2(b) observed (symbols) and predicted values of |〈*v, u*〉|^2^ given by Eq. (10) (curve). Note that the values of |〈*v*^(^*^r^*^)^, *u*^(^*^r^*^)^〉|^2^ depict a phase transition that occurs at a critical subgraph size $K_{r}^{*}$ given by Eq. (11): |〈*v*^(^*^r^*^)^, *u*^(^*^r^*^)^〉|^2^ *>* 0 when $K_{r}>K_{r}^{*}$, whereas |〈*v*, *u*〉|^2^ = 0 when $K_{r}\leq K_{r}^{*}$. This phase transition in eigenvector localization drives a phase transition for community detection based on **v**^(^*^r^*^)^. Arrows indicate the values of *K_r_* used in panel (a).

**FIG. 3:**
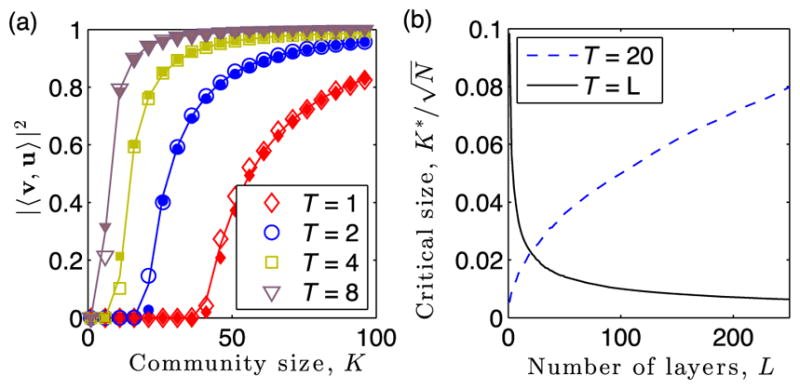
Influence of community persistence *T_r_* on eigenvector localization for summation-based layer aggregation. (a) Observed (symbols) and predicted values of |〈**v**^(^*^r^*^)^, **u**^(^*^r^*^)^〉|^2^ given by Eq. (10) (curves) vs *K_r_* for *T_r_* ∈ {1, 2, 4, 8}. Open symbols indicate the parameters used in Fig. 2, whereas filled symbols indicate when the layers’ edge probabilities {*p_l_*} are drawn uniformly from [0, 0.02]; we plot the mean value of |〈**v**^(^*^r^*^)^, **u**^(^*^r^*^)^〉|^2^ across 10 choices for the sets 𝒦*_r_* and 𝒯 *_r_*. (b) Critical size $K_{r}^{*}$ given by Eq. (11) vs *L* for fixed *T_r_* (dashed line) and *T_r_* = *L* (solid line). As indicated by Eq. (12), layer aggregation by summation can enhance or inhibit detection depending on whether or not the scaling for *T_r_*(*L*) exceeds 𝒪(*L*^1/2^).

In Fig. 3(a), we compare observed (symbols) and predicted values of |〈*v*, *u*〉|^2^ given by Eq. (10) (curves) for varying *K_r_* with *T_r_* ∈ {1, 2, 4, 8}. Open symbols indicate the parameters used in Fig. 2, whereas filled symbols indicate the mean value of |〈**v**, **u**〉|^2^ for 10 trials in which the layers’ edge probabilities {*p_l_*} are drawn uniformly from [0, 0.02]. Note that as *T_r_* increases, the curves shift to the left, illustrating that as the community persists across more layers, the localization phenomenon is stronger and the hidden community is easier to detect. In Fig. 3(b), we study the dependence of $K_{r}^{*}$ on the number of layers, *L*, and we compare the effect of keeping *T_r_* fixed vs allowing *T_r_* to grow with *L*. Specifically, we set either *T_r_*(*L*) = 20 or *T_r_*(*L*) = *L*, and we plot the value of $K_{r}^{*}$ given by Eq. (11). Note that if the community persists across a fraction of the layers—that is, *T_r_*(*L*) = *cL* for some constant c—then $K_{r}^{*}$ vanishes with scaling 𝒪(*L*^−1/2^). However, if *T_r_* is held fixed, then $K_{r}^{*}$ increases with scaling 𝒪(*L*^1/2^).

In summary, these experiments illustrate how layer aggregation through summation can enhance small-community detection if the community persists across sufficiently many layers, but it can obscure detection if the community is present in too few layers. We will see in the next section that thresholding the summation can help overcome this problem, potentially reducing the detectability limit by orders of magnitude to yield super-resolution community detection.

### B. Thresholding as a nonlinear data filter

#### 1. Random matrix theory for modularity matrices

We now study layer aggregation with thresholding as a filter that enhances small-community detection. We begin by solving for *effective* edge probabilities for the thresholding process [10]. Thresholding the summation Σ*_l_***A**^(^*^l^*^)^ at *L̃* yields a binary adjacency matrix **Â**^(^*^L̃^*^)^ with entries ${\hat{A}}_{ij}^{\left(\tilde{L}\right)}\in \{0,1\}$ indicating whether or not *Ā_ij_* ≥ *L̃*. For edges (*i*, *j*)∈∉*_r_*{𝒦*_r_* × 𝒦*_r_*}, *Ā_ij_* follows a Poisson binomial distribution *f*_PB_(*a; L*, {*p_l_*}) given by Eq. (2), and the inequality is satisfied with probability

(13) $${\hat{p}}^{\left(\tilde{L}\right)}=P[{\bar{A}}_{ij}\geq \tilde{L}]=1-F_{\text{PB}}(\tilde{L}-1,L,\{p_{l}\}),$$

where *F*_PB_(*a*, *L*, {*p_l_*}) is the associated cumulative distribution function (CDF). For edges (*i*, *j*) ∈ {𝒦*_r_* × 𝒦*_r_*}, *Ā_ij_* follows a Poisson binomial distribution $f_{\text{PB}}(a;L,\{q_{l}^{\left(r\right)}\})$ given by Eq. (2), and the inequality is satisfied with probability

(14) $${\hat{\rho }}_{r}^{\left(\tilde{L}\right)}=P[{\bar{A}}_{ij}\geq \tilde{L}]=1-F_{\text{PB}}(\tilde{L}-1,L,\{q_{l}^{\left(r\right)}\}),$$

where $q_{l}^{\left(r\right)}=\rho_{r}$ for *l* ∈ 𝒯*_r_* and otherwise $q_{l}^{\left(r\right)}=p_{l}$. In the case of a clique (i.e., *ρ_r_* = 1), Eq. (14) can be written as

(15) $${\hat{\rho }}_{r}^{\left(\tilde{L}\right)}=1-F_{\text{PB}}(\tilde{L}-T_{r}-1,L-T_{r},{\left\{p_{l}\right\}}_{l\notin T_{r}}).$$

Given the effective edge probabilities for the network and a community (i.e., *p̂*^(^*^L̃^*^)^ and ${\hat{\rho }}_{r}^{\left(L\right)}$, respectively), it is straightforward to study the detectability limits of a community for thresholded networks using Eqs. (10) and (11). In particular, we substitute *L* = *T_r_* = 1 to obtain

(16) $${|\langle {\hat{v}}^{\left(r\right)},u^{\left(r\right)}\rangle |}^{2}=\left\{ \begin{array}{ll} 1-1/{\hat{\theta }}_{r}^{2} & {\hat{\theta }}_{r}>1\\ 0 & \text{otherwise}, \end{array}\right.$$

where **v̂**^(^*^r^*^)^ is a dominant eigenvector of modularity matrix

(17) $$\hat{B}={\hat{A}}^{\left(\tilde{L}\right)}-{\hat{p}}^{\left(\tilde{L}\right)}11^{T}$$

and ${\hat{\theta }}_{r}=K({\hat{\rho }}_{r}^{\left(\tilde{L}\right)}-{\hat{p}}^{\left(\tilde{L}\right)})/\sqrt{N{\hat{p}}^{\left(\tilde{L}\right)}(1-{\hat{p}}^{\left(\tilde{L}\right)})}$. Setting *θ̂*_r_ = 1 gives a detectability limit for each community *r* in terms of the effective edge probabilities *p̂*^(^*^L̃^*^)^ and ${\hat{\rho }}_{r}^{\left(\tilde{L}\right)}$,

(18) $${\hat{K}}_{r}^{*}=\frac{\sqrt{N{\hat{p}}^{\left(\tilde{L}\right)}(1-{\hat{p}}^{\left(\tilde{L}\right)})}}{{\hat{\rho }}_{r}^{\left(\tilde{L}\right)}-{\hat{p}}^{\left(\tilde{L}\right)}}.$$

Equations (16)–(18) illustrate that the detectability limits for thresholded networks depend only on the effective edge probabilities; however, these depend sensitively on the choice of threshold *L̃*.

Importantly, ${\hat{K}}_{r}^{*}$ given by Eq. (18) can potentially be orders of magnitude smaller than $K_{r}^{*}$ given by Eq. (11), a phenomenon we call super-resolution detection. In addition to numerical experiments that will follow below, we further study this phenomenon by comparing ${\hat{K}}_{r}^{*}$ and $K_{r}^{*}$ for network parameters wherein we can obtain deeper insight. We consider clique detection (i.e., *ρ_r_* = 1) in a sparse network (i.e., *p_l_* ≪ 1) and focus on the threshold value *L̃* = *T_r_* to obtain

(19) $${\hat{K}}_{r}^{*}\approx \sqrt{N}\sqrt{{\hat{p}}^{\left(T_{r}\right)}}.$$

Using these assumptions also in Eqs. (13) and (15), we find the effective edge probabilities *p̂*^(*T*_*r*_)^=1–*F*_PB_(*T_r_*–1, *L*,{*p_l_*}) and ${\hat{\rho }}_{r}^{\left(T_{r}\right)}=1$. Furthermore, we apply Hoeffding’s inequality [43] to obtain *p̂*^(*T*_*r*_)^ ≤ *e*^−2*L*(〈*p*_*l*_〉−*T*_*r*_/*L*)2^. Noting 0 *<* 〈*p_l_*〉 ≪ *T_r_/L*, we find the 〈*p_l_*〉 → 0 limiting bound

(20) $${\hat{p}}^{\left(T_{r}\right)}\leq e^{-2T_{r}^{2}/L},$$

illustrating that *p̂*^(*T*_*r*_)^ and ${\hat{K}}_{r}^{*}$ decay exponentially with $T_{r}^{2}/L$. On the other hand, we use the sparsity assumption in Eq. (11) to obtain

(21) $$K_{r}^{*}\approx \frac{\sqrt{NL\langle p_{l}\rangle }}{\sqrt{T_{r}^{2}}}.$$

Thus, in this case, $K_{r}^{*}$ decays as $O(1/\sqrt{T_{r}^{2}/L})$, whereas ${\hat{K}}_{r}^{*}$ decays exponentially (i.e., considerably faster) with $T_{r}^{2}/L$.

#### 2. Numerical validation and super-resolution detection

We now support Eqs. (13)–(18) with numerical experiments and illustrate that certain thresholds lead to super-resolution community detection. We consider the detection of a dense subgraph that is hidden in both (a) a dense network with 〈*p_l_*〉 = 0.5 and (b) a sparse network with 〈*p_l_*〉 = 0.01. Both networks were constructed with *N* = 10^4^, *σ_p_* = 0.001, *ρ_r_* = 1, *L* = 16, and *T_r_* = 5.

**FIG. 4:**
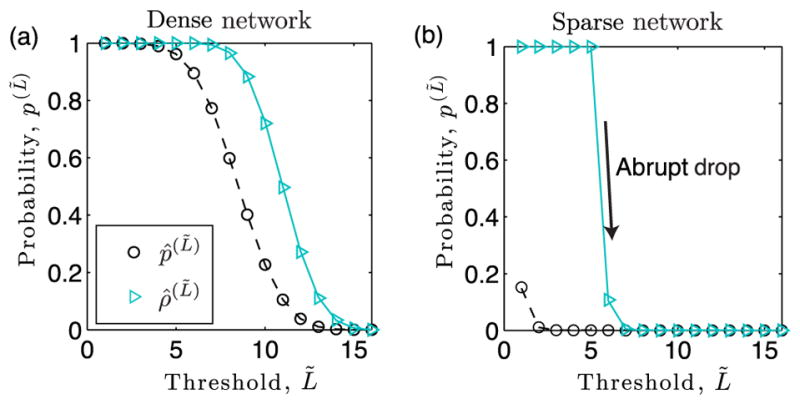
Effective edge probabilities for threshold-based layer aggregation. Observed (symbols) and predicted values given by Eqs. (13) and (15) (curves) for the effective edge probability of the background network, *p̂*^(^*^L̃^*^)^, and for a community, ${\hat{\rho }}_{r}^{\left(\tilde{L}\right)}$, as a function of *L̃*. Network parameters include *N* = 10^4^, *L* = 16, *T* = 5, and *σ_p_* = 0.001 and either (a) 〈*p_l_*〉 = 0.5 or (b) 〈*p_l_*〉 = 0.01. Note that for the sparse network in panel (b), *ρ̂* ^(^*^L̃^*^)^ undergoes an abrupt drop when *L̃* surpasses *T_r_* = 5.

In Fig. 4, we compare observed (symbols) and predicted values (curves) of the effective edge probabilities *p̂*^(^*^L̃^*^)^ given by Eq. (13) and ${\hat{\rho }}_{r}^{\left(\tilde{L}\right)}$ given by Eq. (14) as a function of the threshold *L̃*. Note in both panels that the effective edge probability *p̂* ^(^*^L̃^*^)^ of the background network always decays with increasing *L̃*. In contrast, the effective edge probability between nodes in the community depends on whether or not $\tilde{L}>T_{r}:{\hat{\rho }}_{r}^{\left(\tilde{L}\right)}=1$ when *L̃* ≤ *T_r_* since *ρ* = 1, whereas ${\hat{\rho }}_{r}^{\left(\tilde{L}\right)}$ decays with increasing *L̃* for *L̃ > T_r_*. Importantly, the rate of decay depends on the network’s mean edge density 〈*p_l_*〉: *ρ̂* ^(^*^L̃^*^)^ slowly decreases for the dense network, whereas it abruptly drops for the sparse network.

**FIG. 5:**
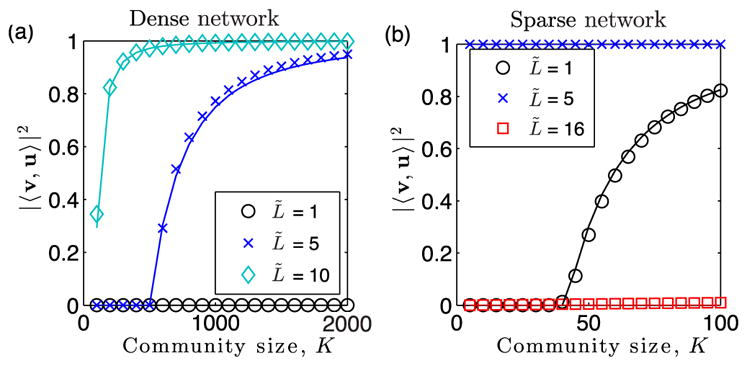
Detectability phase transitions for threshold-based layer aggregation. We plot |〈**v**^(^*^r^*^)^, **u**^(^*^r^*^)^〉|^2^ vs community size *K_r_* with identical parameters to those used to produce Fig. 4 except with selected choices for the threshold *L̃*.

In Fig. 5, we plot observed (symbols) and predicted values (curves) for |〈**v**^(^*^r^*^)^, **u**^(^*^r^*^)^〉|^2^ given by Eq. (16) vs *K* for different choices of *L̃*. The parameters used are identical to those of Fig. 4, and panels (a) and (b) again depict results for 〈*p_l_*〉 = 0.5 and 〈*p_l_*〉 = 0.01, respectively. We highlight several important observations. First, note in both panels that *L̃* = *T_r_* = 5 yields better detectability than *L̃* = 1. However, when *L̃ > T_r_*, we find contrasting results for sparse and dense networks. For the sparse network shown in Fig. 5(b), the hidden community becomes harder to detect when *L̃ > T_r_* (see curve for *L̃* = 16), which intuitively occurs because ${\hat{\rho }}_{r}^{\left(\tilde{L}\right)}$ rapidly decays and the thresholded networks will no longer contain a dense subgraph. On the other hand, for the dense network depicted in Fig. 5(a), increasing *L̃* can improve detectability when *L̃ > T_r_* (see curve for *L̃* = 10).

**FIG. 6:**
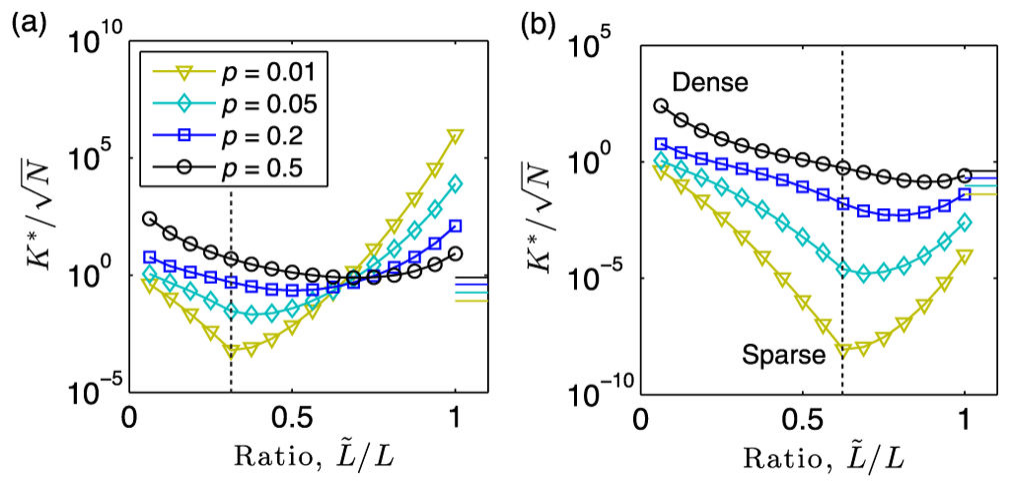
Super-resolution community detection for threshold-based layer aggregation. We plot ${\hat{K}}_{r}^{*}$ given by Eq. (18) as a function of *L̃* for *p* ∈ {0.01, 0.05, 0.2, 0.5}, *N* = 10^4^, *ρ* = 1, *σ_p_* = 0.001, *L* = 16, and either (a) *T_r_* = 5 or (b) *T_r_* = 10. Note that the *L̃* value yielding the minimum ${\hat{K}}_{r}^{*}$ occurs at *L̃* = *T_r_* (vertical dotted lines) for sparse networks, whereas it increases with increasing *p* [e.g., compare *p* = 0.01 and *p* = 0.5 in panel (b)]. The horizontal lines on the right edge of the panels indicate $K_{r}^{*}$ given by Eq. (11) for summation networks. Importantly, thresholding can potentially decrease ${\hat{K}}_{r}^{*}$ by many orders of magnitude as compared to $K_{r}^{*}$.

We now present an experiment highlighting the occurrence of super-resolution community detection for certain threshold values. In Fig. 6, we study the dependence of the critical community size $K_{r}^{*}$ on the threshold *L̃*. We plot ${\hat{K}}_{r}^{*}$ given by Eq. (18) as a function of *L̃* for *p* ∈ {0.01, 0.05, 0.2, 0.5}, *N* = 10^4^, *ρ* = 1, *σ_p_* = 0.001, *L* = 16, and either (a) *T_r_* = 5 or (b) *T_r_* = 10. Note that for the sparsest network, i.e., *p* = 0.01, the minimum value of K_*_ occurs when *L̃* = *T_r_* (vertical dashed line). Interestingly, as the mean edge density *p* = 〈*p_l_*〉 increases, the threshold *L̃* at which ${\hat{K}}_{r}^{*}$ attains its minimum value shifts from *L̃* = *T_r_* towards *L̃* = *L*. The horizontal lines on the right edge of the panels indicate $K_{r}^{*}$ given by Eq. (11) for the summation network.

Importantly, note that for a wide range of parameters, ${\hat{K}}_{r}^{*}$ for the thresholded networks is significantly smaller than $K_{r}^{*}$ for the corresponding summation networks. In particular, one can observe for *p* = 0.1 and *L̃/L* = *T_r_/L* in Fig. 6(b) that ${\hat{K}}_{r}^{*}$ is many orders of magnitude smaller than $K_{r}^{*}\;[O(10^{-6})\;\text{times}\;\text{here}]$. In other words, thresholding the summation can dramatically improve detectability as compared to summation without thresholding. This surprising result contrasts our previous findings for the detectability of large communities that persist across all layers [10], where it was found that thresholding always inhibited detection (although optimal thresholds were found to minimize inhibition).

## IV. SMALL-COMMUNITY DETECTION IN TIME-VARYING NETWORKS

**FIG. 7:**
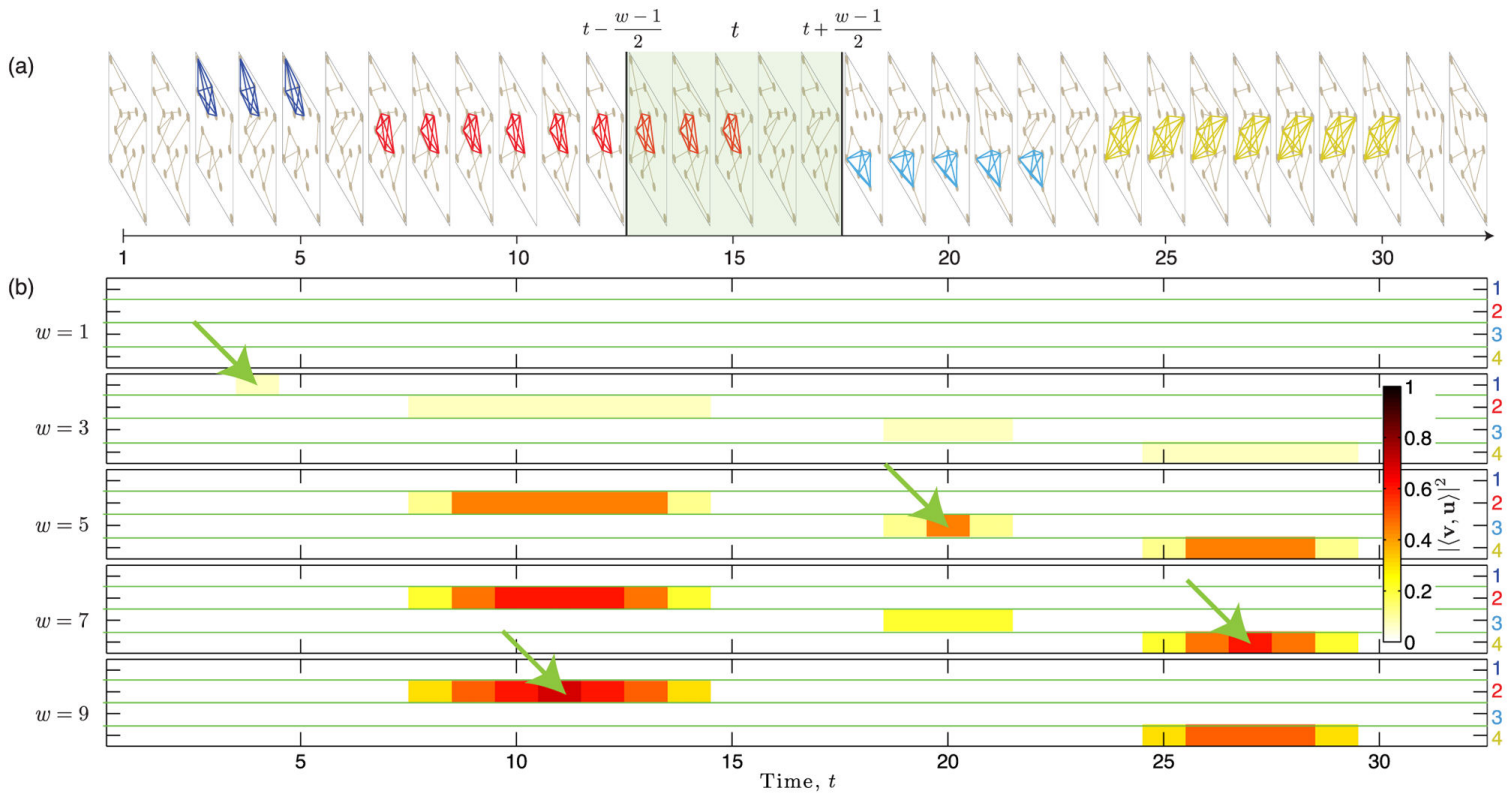
Detectability of small communities in temporal networks with summation-based binning into time windows. (a) Illustration of a temporal network with *L* = 32 time layers and hidden communities that persist across different time layers. The shaded region indicates a bin, or time window, of size *w* ≤ *L* at time *t* for which the layers will be aggregated, which is a process that can be used to discretize and/or smooth the network data. The bin contains layers 𝒲*_w_*(*t*) = {*t* − (*w* − 1)/2,…, *t* + (*w* − 1)/2}. (b) We illustrate by color the values |〈**v**^(^*^r^*^)^, **u**^(^*^r^*^)^〉|^2^ for the aggregation of layers across bins 𝒲*_w_*(*t*) for each of the four communities *r* ∈ {1, 2, 3, 4}. In particular, we show Eq. (10) under the variable substitutions *T_r_*(𝒲*_w_*(*t*)) ↦ *T* and *w* ↦ *L*, where *T_r_*(𝒲*_w_*(*t*)) is the number of layers in which community *r* is present in bin 𝒲*_w_*(*t*). Layer aggregation across each bin was implemented by summation. We study a temporal network with *N* = 10^4^, *L* = 32, *p* = 0.01, *σ_p_* = 0.001, and we show results for several bin widths *w* ∈ {1, 3, 5, 7, 9}. The hidden communities all contain *K_r_* = 8 nodes and have different persistent lengths *T_r_* as depicted in panel (a). The green arrows indicate, for each *r*, the bin location and *w* value at which |〈**v**^(^*^r^*^)^, **u**^(^*^r^*^)^〉|^2^ obtains its maximum.

We now present an experiment involving small-community detection in time-varying networks to highlight several practical insights following from our theoretical results. Note that unlike Sec. III, where there were no restrictions on which layers a community persists, we now assume that each community persists across consecutive layers. We conducted experiments for a synthetic temporal network with *N* = 10^4^ nodes and *L* = 32 time layers, each of which is drawn from an ER network with edge probability *p_l_*, which we drew from a Gaussian distribution with mean *p* = 0.01 and standard deviation *σ_p_* = 0.001. We then planted *R* = 4 communities, each involving *K_r_* = *K* = 8 nodes, in the following sets of layers: 𝒯_1_ = {3, 4, 5} for community 1, 𝒯_2_ = {7,…, 15} for community 2, 𝒯_3_ = {18,…, 22} for community 3, and 𝒯_4_ = {24,…, 30} for community 4. In Fig. 7(a), we provide a representative illustration of the temporal network, where we indicate in which layers the communities are present. We also illustrate by the shaded region an example time window, or bin, 𝒲*_w_*(*t*) = {*t* − (*w* − 1)/2,…, *t* + (*w* − 1)/2} for *t* ∈ {(*w* − 1)/2, *L* − (*w* − 1)/2}, that contains layers to be aggregated.

We first consider aggregation by summation. In Fig. 7(b), we illustrate by color the values |〈**v**^(^*^r^*^)^, **u**^(^*^r^*^)^〉|^2^ for the aggregation of layers across bins 𝒲*_w_*(*t*). In particular, we show Eq. (10) under the variable substitutions *T_r_*(𝒲*_w_*(*t*)) ↦ *T* and *w* ↦ *L*, where *T_r_*(𝒲*_w_*(*t*)) = |𝒲*_w_*(*t*) ∩ 𝒯 *_r_*| is the number of layers in which community *r* is present in bin 𝒲*_w_*(*t*). We show results for several bin widths *w* ∈ {1, 3, 5, 7, 9}. The green arrows indicate, for each *r*, the bin location and *w* value at which |〈**v**^(^*^r^*^)^, **u**^(^*^r^*^)^〉|^2^ obtains its maximum. As expected, |〈**v**^(^*^r^*^)^, **u**^(^*^r^*^)^〉|^2^ obtains its maximum for each community *r* when the bin 𝒲*_w_*(*t*) is exactly the set of layers in which community *r* is present, 𝒲*_w_*(*t*) = 𝒯 *_r_* (i.e., when *T_r_* = *w*).

Before studying aggregation by summation and thresholding, we first make several important observations using Fig. 7. First, note that for *w* = 1 in panel (b), no communities are detectable. In other words, all communities are undetectable if the layers are studied in isolation. However, they can be detected if the layers are binned into time windows. Second, because the optimal bin size *w* is unique to every community (i.e., because they have different persistence *T_r_* ∈ [3, 9]), there is no bin size that is best for all communities. In fact, detectability requires $K_{r}>K_{r}^{*}$ given by Eq. (11), which requires that, for each community, *w* is not too large or too small. For example, community 1 is only detectable when *w* = 3, and community 3 is only detectable when *w* ∈ [3, 7].

One final important observation for Fig. 7(b) is that even when communities are detectable, the values |〈**v**^(^*^r^*^)^, **u**^(^*^r^*^)^〉|^2^ are not very large—specifically, |〈**v**^(^*^r^*^)^, **u**^(^*^r^*^)^〉|^2^ ≤ 0.7 in all cases. This can be problematic since detection error rates increase as |〈**v**^(^*^r^*^)^, **u**^(^*^r^*^)^〉|^2^ decreases, approaching 100% error as |〈**v**^(^*^r^*^)^, **u**^(^*^r^*^)^〉|^2^ → 0. (See Ref. [27] for an analysis of error rates based on a hypothesis-testing framework for clique detection in single-layer networks.) Because |〈**v**^(^*^r^*^)^, **u**^(^*^r^*^)^〉|^2^ remains small for community 1 for all choices of *w*, it effectively remains undetectable by summation-based layer aggregation.

**FIG. 8:**
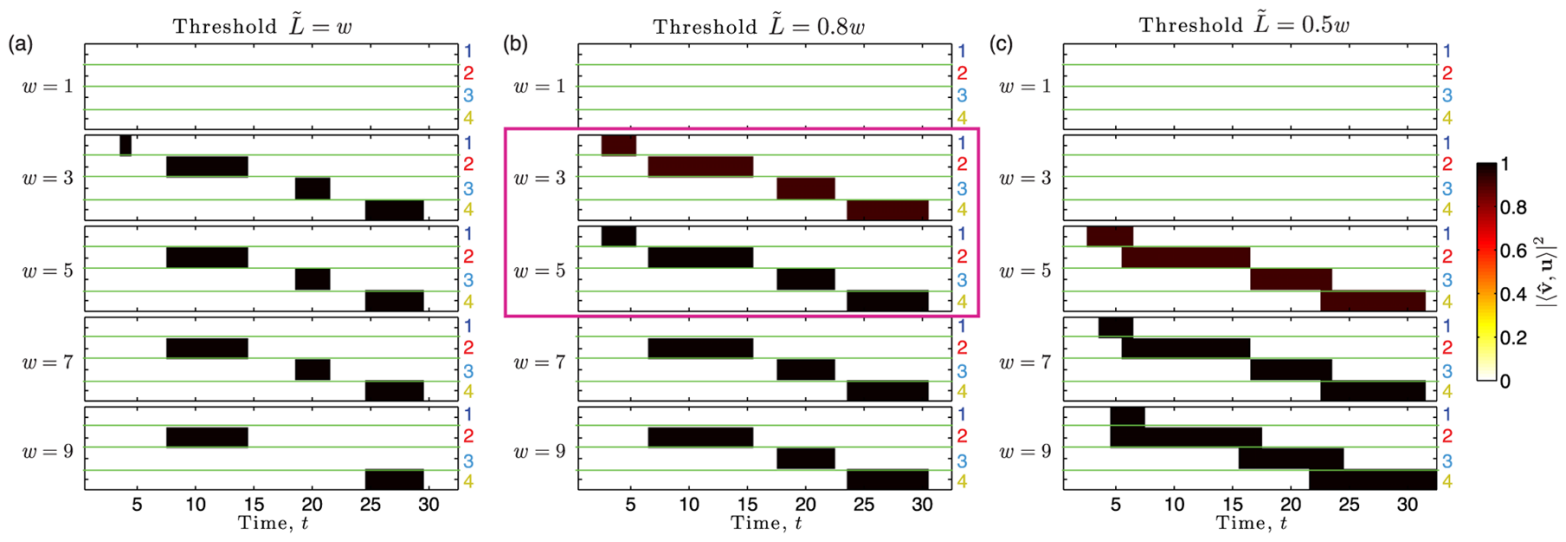
Detectability of small communities in temporal networks with time-window binning by summation and thresholding. We illustrate by color the values |〈**v̂**^(^*^r^*^)^, **u**^(^*^r^*^)^〉|^2^ given by Eq. (16) for each of the four communities *r* ∈ {1, 2, 3, 4} with the variable substitutions *T_r_*(𝒲*_w_*(*t*)) ↦ *T* and *w* ↦ *L* into Eqs. (13)–(18). Results are shown for bins of width *w* ∈ {1, 3, 5, 7, 9} for a temporal network with *N* = 10^4^ nodes, *L* = 32 time layers, and hidden communities as depicted in Fig. 7(a). The communities each contain *K_r_* = *K* = 8 nodes and have different persistence lengths *T_r_*. Layer aggregation across each bin was implemented by summation and thresholding at *L̃*. Panels (a)–(c) respectively indicate the choices *L̃* = *w*, *L̃* = 0.8*w*, and *L̃* = 0.5*w*. The violet box in panel (b) indicates combinations of thresholds and bin sizes that yield accurate detection of all four communities. We stress, however, that since the detectability-limit criterion given by Eq. (18) depends on a complex interplay between the community and network characteristics, one should not, in general, expect there to exist a *single* best combination for *all* communities.

We now illustrate layer aggregation with thresholding as a filter that can allow greatly improved small-community detection for the temporal network shown in Fig. 7(a), including the accurate recovery of community 1. In Fig. 8, we plot |〈**v̂**^(^*^r^*^)^, **u**^(^*^r^*^)^〉|^2^ given by Eq. (16) with the variable substitutions *T_r_*(𝒲*_w_*(*t*)) ↦ *T* and *w* ↦ *L* into Eqs. (13)–(18). Results reflect the aggregation of layers into bins 𝒲*_w_*(*t*) for each of the four communities *r* ∈ {1, 2, 3, 4} and with bin sizes *w* ∈ {1, 3, 5, 7, 9}. Panels (a)–(c) indicate results for different thresholds, *L̃* ∈ {*w*, 0.8*w*, 0.5*w*}.

Our first observation for Fig. 8 is that none of the communities can be detected (for any threshold) if the layers are analyzed in isolation (see results for window size *w* = 1). This result is similar to that shown in Fig. 7(b) for summation without thresholding (i.e., whenever *w* = 1, we find |〈**v̂**^(^*^r^*^)^, **u**^(^*^r^*^)^〉|^2^ = |〈**v**^(^*^r^*^)^, **u**^(^*^r^*^)^〉|^2^ = 0). In other words, the detectability of communities is only made possible through layer aggregation.

Our next observation is that the values |〈**v̂**^(^*^r^*^)^, **u**^(^*^r^*^)^〉|^2^ are either zero or close to one, which is in sharp contrast to the values of |〈**v**^(^*^r^*^)^, **u**^(^*^r^*^)^〉|^2^ shown in Fig. 7(b), which can be observed to obtain many values across the range [0, 0.7]. In other words, in this experiment, the use of thresholding as a filter allows small communities to be either strongly detected or not detected—there is no middle ground for weak detection (which is the case for layer aggregation without thresholding). This is important since error rates for community detection vanish as |〈**v̂**^(^*^r^*^)^, **u**^(^*^r^*^)^〉|^2^ → 1 [27].

Our final observation is that different threshold values enhance the detectability of different communities. For example, community 1 is detectable when *w* = 3 for *L̃* ≥ 0.8*w* but not for *L̃* = 0.5*w* [compare panels (a) and (b) to panel (c)]. Similarly, community 3 is detectable when *w* = 9 for *L̃* ≤ 0.8*w* but not for *L̃* = *w* [compare panels (b) and (c) to panel (a)]. Interestingly, in this experiment, we were able to identify a combination of parameters (*L̃*,*w*) that allows accurate detection of all four communities—that is, |〈**v̂**^(^*^r^*^)^, **u**^(^*^r^*^)^〉|^2^ ≈ 1 for bin 𝒲*_w_*(*t*) only when community *r* is present in time layer *t* [i.e., *t* ∈ 𝒯*_r_*]; otherwise, |〈**v̂**^(^*^r^*^)^, **u**^(^*^r^*^)^〉|^2^ ≈ 0. We highlight these values of ( *L̃*,*w*) in panel (b) with a violet box. However, we stress that these “best” values for ( *L̃*, *w*) arise in this experiment because the communities are relatively similar in size (i.e., *K_r_* ∈ [3, 9]) and density (i.e., *ρ_r_* = 1). In general, one should not expect there to exist one choice of parameters ( *L̃*,*w*) to work well for all communities since the detectability-limit criterion given by Eq. (18) depends on a complex interplay between the network and community parameters {*p_l_*}, *ρ_L_*, *T_r_*, *K_r_*, *L*, and *L̃*.

## V. DISCUSSION

There is considerable need to better understand how network preprocessing affects network-analysis methodologies. Herein, we studied how different methods for layer aggregation affect the detectability of small-scale communities in multilayer networks (including multilayer representations of temporal networks). Small-community detection is widely used for anomaly detection in network data [23–28]; in cybersecurity, for example, it allows detection of harmful events such as attacks [23], intrusions [24], and fraud [25]. Understanding limitations on small-community detection provides insight towards the detectability of these harmful activities. Despite most networks inherently changing in time, previous theory for limitations on small-community detection have been restricted to single-layer networks [26,27] or summation-based aggregation [11]. We highlight that our model and analysis generalizes these previous works in several ways: (i) A community has edge probability *ρ* ∈ (0, 1] and is not necessarily a clique, (ii) a community can persist across a subset of layers, (iii) the mean edge probability *p_l_* can vary across network layers, and (iv) the multilayer or temporal network can simultaneously contain several communities.

Motivated in this way, we developed random matrix theory [27,40] to analyze detectability phase transitions in which the dominant eigenvectors of modularity matrices associated with layer-aggregated multilayer networks localize onto communities, thereby allowing their detection. We developed theory for when a community with *K_r_* ≪ *N* nodes is hidden (i.e., planted) in *T_r_* ≤ *L* layers of a multilayer network with *N* nodes and *L* layers. We found a detectability phase transition to occur for a given community *r* when its size *K_r_* surpasses a detectability limit. When layers are aggregated by summation, the detectability limit $K_{r}^{*}$ is given by Eq. (11) and has the scaling behavior $K_{r}^{*}\propto \sqrt{NL}/T_{r}$. Surprisingly, if *L* is allowed to vary, this implies that summation-based aggregation enhances community detection even if the community exists in a vanishing fraction *T_r_/L* of layers, provided that *T_r_/L* decays more slowly than 𝒪(*L*^−1/2^). This result is surprising since layer aggregation still benefits community detection despite the fact that most layers carry no information about the community.

We also introduced and studied the utility of layer aggregation with thresholding as a nonlinear data filter to enhance small-community detection. Our analysis [particularly, Eq. (18)] revealed that in addition to implementing sparsification and dichotomization, thresholding can allow super-resolution community detection, whereby the detectability limit decreases by several orders of magnitude (see Fig. 6). In particular, we showed in Sec. III B that ${\hat{K}}_{r}^{*}$ decays exponentially with $\sqrt{L}/T_{r}$ for clique detection in layer-aggregated sparse networks filtered by threshold *L̃* = *T_r_*.

To illustrate practical implications of our results, in Sec. IV we presented an experiment involving the detection of small communities in a time-varying network, highlighting the following key insights:

- Aggregating time layers into appropriate-sized bins can allow the detection of small communities that would otherwise be undetectable (that is, if the layers were considered in isolation or if all layers were aggregated).
- Layer aggregation by summation enhances community detection if the community persists across sufficiently many [specifically, 𝒪(*L*^1/2^)] layers; otherwise, it can obscure detection.
- Layer aggregation with thresholding is a filter that can allow super-resolution community detection of small communities that are otherwise too small for detection.
- The threshold that best enhances the detection of a small community depends on many parameters, and the detection of multiple communities should, in general, utilize multiple thresholds.

We have thus provided a theoretical framework supporting how small-community detection in temporal network data can be improved through network preprocessing in which network layers are binned into time windows and are aggregated using summation with thresholding. This filtering, however, should not be approached as a “one-size-fits-all” procedure. In particular, we find that there exist optimal time window sizes *w* and layer-aggregation strategies that, in general, are unique to each community (i.e., depending on its size, density, persistence across the layers, etc.). While it is important to consider a range of window sizes and layer-aggregation methods, this leads to an unavoidable trade-off between computational cost and sufficient exploration of different parameters.

Before concluding, we discuss implications of our work regarding the topic of eigenvector localization in complex networks, which is an important topic in network science [44,45] for the study of centrality [46–48], spatial analysis [49], and core-periphery structure [50,51]. In particular, there is growing interest in extending these ideas to time-varying [52] and multilayer networks [53]. Recently, Ref. [54] showed that an Anderson-localization-type transition occurs for material transport on several real-world networks (e.g., interconnected ponds of melting sea ice, porous human bone, and resistor networks) and noted that they did not observe the wave interference and scattering effects that typically occur for Anderson localization (a widely studied phenomenon in which eigenfunctions localize onto defects in disordered materials [55,56]). Reference [54] found the phase transition to coincide with a phase transition in network connectivity due to eigenvector localization onto different connected components. Our work complements these findings, showing that a similar localization phenomenon can be brought on by small communities—that is, localization does not necessarily require network fragmentation. (We note in passing that connected components can be interpreted as one, and perhaps the strictest, notion of a community.) Future research should further explore the connection between community-based and connected-component-based eigenvector localization on networks, and their relationship to Anderson localization in materials. (See Refs. [57,58] for related research using network-based models for disordered and composite materials.)

Finally, we highlight other extensions to our work that would be interesting to pursue. Motivated by applications for data fusion, recent research [11] considered weighted averaging of adjacency matrices, allowing them to optimize the weights for the different network layers. It would be interesting to extend our research to weighted averages, which should be fairly straightforward by redefining 〈·〉 in Eqs. (9)–(11) with weights. We leave open the joint optimization of weighting and thresholding. Finally, it would also be interesting to use our method to study the temporal behavior of communities [59], such as a set of nodes that form a recurring community in different time windows (i.e., periodically or stochastically).
